# Attitudes and Beliefs Associated With COVID-19 Vaccination During Pregnancy

**DOI:** 10.1001/jamanetworkopen.2022.7430

**Published:** 2022-04-14

**Authors:** Yiwen Cui, Kole Binger, Anna Palatnik

**Affiliations:** 1 Division of Maternal Fetal Medicine, Department of Obstetrics and Gynecology, Medical College of Wisconsin, Milwaukee; 2 Medical College of Wisconsin, Milwaukee

## Abstract

This survey study examines the attitudes and beliefs associated with receipt of the COVID-19 vaccination during pregnancy among pregnant people.

## Introduction

Pregnant people with SARS-CoV-2 infection have obstetric and medical complications during and after pregnancy.^1^ Receiving COVID-19 vaccine reduces these complications by 90%.^2^ However, vaccine uptake in pregnancy remains low. As of January 15, 2022, only 42.6% of pregnant people were fully vaccinated, with 24.4% receiving at least 1 vaccine dose during pregnancy.^3^ To address this public health challenge, we designed and validated a survey to identify strategies to address vaccine-hesitancy in pregnant people. The objective of this study was to investigate which maternal self-reported attitudes and beliefs are associated with COVID-19 vaccine uptake during pregnancy.

## Methods

This prospective survey study of unvaccinated pregnant people was conducted at a single tertiary care center between June and August 2021 and followed the American Association for Public Opinion Research (AAPOR) reporting guideline. Medical College of Wisconsin institutional review board approval was obtained prior to all study procedures. Electronic informed consent was obtained from all eligible participants. The survey included sociodemographic questions and items focused on general immunization practice, attitudes toward SARS-CoV-2, and concerns regarding safety of the COVID-19 vaccine. The survey was validated by our team and demonstrated internal consistency with Cronbach α of 0.50 to 0.77 (see eAppendix in the Supplement). COVID-19 vaccination, defined as receipt of at least 1 dose of the vaccine during pregnancy, was abstracted from medical records after pregnancy completion. Statistical analyses were performed in R version 4.1.0 (R Project for Statistical Computing). The survey questions are in the eTable in the Supplement.

## Results

Among 295 unvaccinated pregnant respondents who completed the survey during the study period, the mean (SD) age was 30.8 (5.2) years; 28 (9.5%) were Hispanic, 47 (15.9%) were non-Hispanic Black, and 219 (74.2%) were non-Hispanic White; and 167 (56.6%) received COVID-19 vaccine by the end of pregnancy. Respondents who ultimately got the vaccine were more likely to self-identify as non-Hispanic White, be married, and have higher education and household income (Table 1). They also had a higher proportion of favorable behaviors of general vaccination including acceptance of vaccination for their children and receipt of annual influenza vaccine for themselves compared with the unvaccinated group.

**Table 1.** Maternal Demographics and General Vaccine Practices

Characteristic	Respondents, No. (%)		*P* value
	Vaccinated (n = 167)	Unvaccinated (n = 128)	
Maternal age, mean (SD), y	31.4 (4.9)	30.4 (5.5)	.11
Gestational age at the time of survey intake, median (IQR), wk	17.0 (15.6-25.9)	21.9 (15.9-25.9)	<.001
Parity, mean (SD)	2.1 (2.5)	2.2 (2.2)	.80
No. of prior miscarriages, mean (SD)	1.6 (0.5)	1.5 (0.5)	.09
Hispanic ethnicity	13 (7.8)	15 (11.7)	.31
Race			
Non-Hispanic Black	17 (10.2)	30 (23.4)	<.001
Non-Hispanic White	138 (82.6)	81 (63.3)	
Other (Asian, Native Hawaiian, or multiracial)	12 (7.2)	17 (13.3)	
Marital status			
Single	28 (16.8)	48 (37.5)	<.001
Married	139 (83.2)	76 (59.4)	
Separated	0	4 (3.3)	
Highest level of education (missing n = 2)			
<High school degree	2 (1.2)	3 (2.3)	<.001
Graduate equivalency degree	10 (6.1)	28 (21.9)	
Some college	20 (12.1)	40 (31.2)	
4-y college	57 (34.5)	33 (25.8)	
>4-y college	76 (46.1)	24 (18.8)	
Household income (missing n = 3), $			
<30 000	18 (10.9)	30 (23.6)	<.001
30 000-50 000	18 (10.9)	28 (22.0)	
50 000-75 000	19 (11.5)	19 (15.0)	
>75 000	110 (66.7)	50 (39.4)	
History of COVID-19 infection	28 (17.0)	25 (19.5)	.65
General vaccine attitude (missing n = 5)			<.001
Agree that vaccines are good	149 (91.4)	89 (70.1)	<.001
Undecided	3 (1.8)	26 (20.5)	
Disagree	11 (6.7)	12 (9.4)	
General vaccine practices			
Ever received in pregnancy	104 (62.3)	55 (42.9)	<.001
Ever declined in pregnancy	25 (14.9)	53 (41.4)	<.001
Ever declined for children	4 (2.4)	22 (17.2)	.002
How often to accept the flu vaccine (missing n = 5)			
Yearly	118 (72.4)	51 (40.2)	<.001
Often	17 (10.4)	13 (10.2)	
Rarely	19 (11.7)	38 (29.9)	
Never	9 (5.5)	25 (19.7)	

Table 2 describes respondents’ attitudes and beliefs about COVID-19 vaccine and SARS-CoV-2 infection. A higher percentage of unvaccinated respondents had concerns over long-term vaccine effects and research to support vaccination in pregnancy. Both groups were divided on vaccine efficacy: 57 vaccinated respondents (34.1%) and 17 unvaccinated respondents (13.3%) believed that the vaccine would protect against personal infection (*P* = .003). However, a larger proportion of the vaccinated group believed in the ability of the vaccine to protect family members (134 vaccinated respondents [80.2%] vs 54 unvaccinated respondents [42.2%]; *P* < .001) and to pass immunity to the infant (103 vaccinated respondents [61.7%] vs 42 unvaccinated respondents [32.8%]; *P* < .001). In a multivariate regression model, the belief that the vaccine will pass immunity to the infant significantly increased the odds for vaccination (adjusted odds ratio [aOR], 3.82; 95% CI, 1.33-11.00), whereas having concerns about long-term effects of the vaccine remained a significant factor against vaccination (aOR, 0.28; 95% CI, 0.11-0.74).

**Table 2.** Attitudes and Beliefs Associated With COVID-19 Vaccination During Pregnancy

Attitudes and beliefs	Participants who answered yes to the question asked		Agree (reference: disagree)		
	No. (%)		OR (95% CI)		
	Vaccinated (n = 167)	Unvaccinated (n = 128)	Model 1^a^	Model 2^b^	Model 3^c^
COVID-19 vaccine concerns					
Not enough is known about long-term effects	61 (36.5)	80 (62.5)	0.22 (0.11-0.43)	0.28 (0.12-0.61)	0.28 (0.11-0.74)
Not enough research to support vaccine inpregnancy	64 (38.3)	83 (64.8)	0.26 (.19-0.58)	0.36 (0.17-0.75)	0.42 (0.18-1.01)
COVID-19 vaccine social attitudes					
Getting the vaccine will protect me against COVID-19	57 (34.1)	17 (13.3)	3.20 (1.61-6.36)	2.16 (0.98-4.78)	1.79 (0.71-4.54)
Getting the vaccine will help protect my family	134 (80.2)	54 (42.2)	5.47 (2.56-11.71)	3.70 (1.52-9.01)	3.00 (0.97-9.25)
Getting the vaccine will pass immunity to my baby	103 (61.7)	42 (32.8)	5.94 (2.82-12.53)	3.85 (1.56-9.50)	3.82 (1.33-11.00)
SARS-CoV-2 infection attitudes					
COVID-19 is a serious disease	156 (93.4)	98 (76.6)	2.68 (0.85-8.45)	1.77 (0.45-6.28)	2.08 (0.47-9.17)
Pregnant patients with COVID-19 are more likely to have more severe disease than nonpregnant patients	131 (78.4)	61 (47.7)	1.97 (0.86-4.52)	1.37 (0.52-3.61)	0.78 (0.24-2.53)

^a^ Model 1 was the unadjusted model.

^b^ Model 2 was adjusted for gestational age, race, education, marital status, and income.

^c^ Model 3 was adjusted for gestational age, race, education, marital status, income, general vaccine attitude, general vaccine practices (ever received vaccine during pregnancy, ever declined vaccine in pregnancy, ever declined vaccine for children and frequency of flu vaccine).

## Discussion

Our study identified 2 beliefs directly associated with COVID-19 vaccine uptake during pregnancy: (1) concerns about long-term effects of the vaccine and (2) belief in the ability of the vaccine to pass immunity to the infant. Our findings highlight important factors that could be targeted by interventions to address vaccine hesitancy in pregnant people. For example, local and national health campaigns can provide real-time access of pregnant people and their health care clinicians to the growing scientific evidence demonstrating transplacental and breastmilk antibody transfer following COVID-19 vaccination.^4,5,6^ It is also important to continue research maintaining vaccination in pregnancy registries and publish data on long-term health outcomes of vaccinated pregnant people and their infants. The limitations of this study include low representation of racial groups other than Black and White, and the results may not be representative of other US regions, and a cross-sectional survey design with assessment of attitudes toward COVID-19 vaccination only once during pregnancy.

## Notes

Supplement. **eAppendix.** Study Design **eTable.** COVID-19 Vaccine Attitudes in Pregnancy Survey Questions
eReferences
