# Supplementary material for: Attitudes and Beliefs Associated With COVID-19 Vaccination During Pregnancy
Source: JAMA Netw Open. 2022 Apr 14;5(4):e227430. doi: 10.1001/jamanetworkopen.2022.7430 (PMC9011126; doi:10.1001/jamanetworkopen.2022.7430)
Supplement: Supplement. — eAppendix. Study Design eTable. COVID-19 Vaccine Attitudes in Pregnancy Survey Questions eReferences [file jamanetwopen-e227430-s001.pdf]

Supplemental Online Content

Cui Y, Binger K, Palatnik A. Attitudes and beliefs associated with COVID-19 vaccination during pregnancy. *JAMA Netw Open.* 2022;5(4):e227430. doi:10.1001/jamanetworkopen.2022.7430

eAppendix. Study Design
eTable. COVID-19 Vaccine Attitudes in Pregnancy Survey Questions
eReferences

This supplemental material has been provided by the authors to give readers additional information about their work.

© 2022 Cui Y et al. *JAMA Network Open.*

eAppendix. Study design

Prior to study initiation, an approval for the protocol was obtained from the Institutional Review Board of the Medical College of Wisconsin. The questionnaire was adapted from a previously validated survey to identify vaccine-hesitant parents[1-4] and modified based on clinical experience and existing literature with the goal of obtaining information specifically focused on COVID-19 vaccine hesitancy and attitudes regarding COVID-19 vaccination during pregnancy. The primary outcome of the COVID-19 Vaccine Attitudes in Pregnancy survey was the respondent's answer on a 10-point scale measuring strength of intention to receive the COVID-19 vaccine during pregnancy. In addition to questions relating to sociodemographic characteristics, the survey contained 17 items (Supplemental Table 1) from 4 domains: general immunization practices, attitudes toward SARS-CoV-2, concerns about the COVID-19 vaccine, and social and informational attitudes toward the vaccine. Three response formats were used: dichotomous responses (e.g., yes/no), 5-point Likert scale (e.g., strongly disagree, disagree, not sure, agree, strongly agree), and a 10-point scale (e.g., from "1– strongly disagree" to "10 – strongly agree"). Factor analysis was used to determine the optimal number of grouping survey items under a shared broader construct.[5] Initial grouping was done by linking conceptually similar areas of questioning. Survey items were then loaded on the identified factors. Internal consistency within each factor was tested with Cronbach's alpha coefficient and internal consistency was found to be moderate (Cronbach's alpha 0.50-0.77, Supplemental Table 2). Survey items related to COVID-19 vaccine hesitancy were tested for association with the intentions of COVID-19 vaccination. The question "ever had a bad reaction to vaccines" was removed from subsequent analyses due to insignificant statistical significance by Spearman's correlation (Supplemental Table 2). Factor analysis confirmed that the five-factor solution provided the best fit for the data (p=0.703, factor loadings >0.3). The survey took less than 10 minutes to complete and read at a 6th grade level. Informed consent was obtained from all study participants prior to taking the survey. Race was self-reported by the survey participants. Due to insufficient sample size to allow meaningful analyses, Asian, Native Hawaiian, or multiracial self-reported race was combined into "other" category.

© 2022 Cui Y et al. *JAMA Network Open.*

eTable: COVID-19 Vaccine Attitudes in Pregnancy survey questions

Variables	Abbreviated item wording	Response categories<br>(# of scale points)
Outcome		
Likelihood	How likely are you to get the vaccine in pregnancy	Very unlikely (1) – Very likely (10)
General immunization practice		
Vaccine behavior (pregnancy)	Ever received a vaccine in pregnancy?	Yes or No
Vaccine behavior (pregnancy)	Ever declined a vaccine in pregnancy?	Yes or No
Decision for children	Ever declined a vaccine for your children?	Yes or No
Vaccine reaction	Ever had a bad reaction to vaccines?	Yes or No
Flu vaccine behavior	How often do you get flu vaccine?	Yearly (1) – Never (4)
Vaccine attitude	Do you believe vaccine is good?	Strongly agree (1) – Strongly disagree (5)
SARS-CoV-2 attitude		
Serious infection	Do you think it is a serious infection	Strongly agree (1) – Strongly disagree (5)
Pregnancy outcome	Do you agree with pregnant women with the infection get sicker than nonpregnant persons?	Strongly agree (1) – Strongly disagree (5)
COVID-19 vaccine concern		
Effects	Not enough known about long-term effects of the vaccine?	Strongly disagree (1) – strongly agree (10)
Research	Not enough research is there to support vaccination in pregnancy?	Strongly disagree (1) – strongly agree (10)
Reduction	What would reduce your concern the most?	Personally knowing a pregnant person who received the vaccine<br>Seeing other pregnant women receiving the vaccine<br>Physician recommendation<br>CDC recommendation<br>Published data<br>Community leader recommendation<br>Financial incentives<br>Access to vaccine in prenatal visit<br>Nothing will reduce my concern<br>I have no concern<br>Multiple of above
COVID-19 vaccine social attitudes		
Protect (me)	The vaccine will protect me	Strongly disagree (1) – strongly agree (10)

© 2022 Cui Y et al. *JAMA Network Open.*

Protect (family)	The vaccine will protect my family	Strongly disagree (1) – strongly agree (10)
Passive immunity	The vaccine will pass immunity to my baby	Strongly disagree (1) – strongly agree (10)
Informational attitude		
Source	Where do you get the most information about the vaccine	Social media<br>Newspaper<br>Television<br>Word-of-mouth<br>Physician
Physician trust	I can openly discuss vaccine concerns with OBGYN	Strongly agree (1) – Strongly disagree (5)
Action	If your OBGYN recommends the vaccine, would you get it?	Yes (1), Undecided (2), No (3)

© 2022 Cui Y et al. *JAMA Network Open.*

eReferences

1. Malik AA, McFadden SM, Elharake J and Omer SB. Determinants of COVID-19 vaccine acceptance in the US. *EClinicalMedicine* 2020;26:100495. doi: 10.1016/j.eclinm.2020.100495.
2. Opel DJ, Taylor JA, Mangione-Smith R, et al. Validity and reliability of a survey to identify vaccine-hesitant parents. *Vaccine* 2011;29(38):6598-605. doi: 10.1016/j.vaccine.2011.06.115.
3. Opel DJ, Mangione-Smith R, Taylor JA, et al. Development of a survey to identify vaccine-hesitant parents the parent attitudes about childhood vaccines survey. *Hum Vaccin* 2011;7(4):419-25. doi: 10.4161/hv.7.4.14120.
4. Opel DJ, Taylor JA, Zhou C, Catz S, Myaing M, Mangione-Smith R. The relationship between parent attitudes about childhood vaccines survey scores and future child immunization status: a validation study. *JAMA Pediatr* 2013;167(11):1065-71. doi: 10.1001/jamapediatrics.2013.2483.
5. Costello AB, Osborne J. Best practices in exploratory factor analysis: four recommendations for getting the most from your analysis. *Practical Assessment, Research and Evaluation* 2005;10:1-9. doi: https://doi.org/10.7275/jyj1-4868.

© 2022 Cui Y et al. *JAMA Network Open.*
